# Supplementary material for: A scoping review on control strategies for Echinococcus granulosus sensu lato
Source: medRxiv. 2024 Aug 21:2024.08.21.24312335. Preprint. [Version 1] doi: 10.1101/2024.08.21.24312335 (PMC11370509; doi:10.1101/2024.08.21.24312335)
Supplement: Supplement 1 [file NIHPP2024.08.21.24312335v1-supplement-1.pdf]

medRxiv preprint doi: https://doi.org/10.1101/2024.08.21.24312335; this version posted August 21, 2024. The copyright holder for this preprint (which was not certified by peer review) is the author/funder, who has granted medRxiv a license to display the preprint in perpetuity.
It is made available under a CC-BY 4.0 International license .

Supporting information

Appendix 1. Search Strategy

Search Concepts

Echinococcus Granulosus	Strategies
Echinococcus	Prevention
Echinococcosis	Control
Echinococcoses	Preventive therapy
Echinococcus Infection	Preventive measures
Echinococcus Infections	Prophylaxis
Infection, Echinococcus	Therapeutic
Cystic Echinococcosis	Therapy
Cystic Echinococcoses	Treatment
Hydatidosis	Treatments
Hydatidoses	Elimination
Hydatid Cysts	Intervention
Hydatid Cyst	Early intervention
Hydatid Disease	Late intervention
Hydatid Diseases	Drug therapy
Echinococcus Granulosus Infection	Drug Therapies
	Pharmacotherapy

medRxiv preprint doi: https://doi.org/10.1101/2024.08.21.24312335; this version posted August 21, 2024. The copyright holder for this preprint (which was not certified by peer review) is the author/funder, who has granted medRxiv a license to display the preprint in perpetuity.
It is made available under a CC-BY 4.0 International license .

	Pharmacotherapies
	Vaccine
	Health Promotion
	Health Campaign
	Pest control
	Pest management

MEDLINE Search strategy:

1. Echinococcus Granulosus
2. Echinococcus
3. Echinococcosis
4. Echinococcoses
5. Echinococcus Infection
6. Echinococcus Infections
7. Infection, Echinococcus
8. Cystic Echinococcosis
9. Cystic Echinococcoses
10. Hydatidosis
11. Hydatidoses
12. Hydatid Cysts
13. Hydatid Cyst
14. Hydatid Disease
15. Hydatid Diseases
16. Echinococcus Granulosus Infection
17. 1-16 OR
18. Strategies
19. Prevention

medRxiv preprint doi: https://doi.org/10.1101/2024.08.21.24312335; this version posted August 21, 2024. The copyright holder for this preprint (which was not certified by peer review) is the author/funder, who has granted medRxiv a license to display the preprint in perpetuity.
It is made available under a CC-BY 4.0 International license .

20. Control
21. Preventive therapy
22. Preventive measures
23. Prophylaxis
24. Therapeutic
25. Therapy
26. Treatment
27. Treatments
28. Elimination
29. Intervention
30. Early intervention
31. Late intervention
32. Drug therapy
33. Drug Therapies
34. Pharmacotherapy
35. Pharmacotherapies
36. Vaccine
37. Health Promotion
38. Health Campaign
39. Pest control
40. Pest management
41. 18-40 OR
43. 17 AND 41

For MEDLINE and EMBASE we used MeSH subject heading.

Search terms used are centered on the concepts of ‘*Echinococcus granulosus* infection’ and ‘strategy’. The ‘*Echinococcus granulosus*’ concept was built around synonyms (e.g., Hydatid disease, Hydatid cyst). The “strategy” concept was broad and included terms covering all types of interventions (e.g., Pest control, Therapy, Prevention). Qualified academic librarian support was requested for identification of key words on the different databases and search strategy refinement.

medRxiv preprint doi: https://doi.org/10.1101/2024.08.21.24312335; this version posted August 21, 2024. The copyright holder for this preprint (which was not certified by peer review) is the author/funder, who has granted medRxiv a license to display the preprint in perpetuity.
It is made available under a CC-BY 4.0 International license .

Appendix 2. Preferred Reporting Items for Systematic reviews and Meta-Analyses extension for Scoping Reviews (PRISMA-ScR) Checklist

SECTION	ITEM	PRISMA-ScR CHECKLIST ITEM	REPORTED ON PAGE #
**TITLE**			
Title	1	Identify the report as a scoping review.	1
**ABSTRACT**			
Structured summary	2	Provide a structured summary that includes (as applicable): background, objectives, eligibility criteria, sources of evidence, charting methods, results, and conclusions that relate to the review questions and objectives.	2
**INTRODUCTION**			
Rationale	3	Describe the rationale for the review in the context of what is already known. Explain why the review questions/objectives lend themselves to a scoping review approach.	3-4
Objectives	4	Provide an explicit statement of the questions and objectives being addressed with reference to their key elements (e.g., population or participants, concepts, and context) or other relevant key elements used to conceptualize the review questions and/or objectives.	5-6
**METHODS**			
Protocol and registration	5	Indicate whether a review protocol exists; state if and where it can be accessed (e.g., a Web address); and if available, provide registration information, including the registration number.	5
Eligibility criteria	6	Specify characteristics of the sources of evidence used as eligibility criteria (e.g., years considered, language, and publication status), and provide a rationale.	6-7, 57
Information sources*	7	Describe all information sources in the search (e.g., databases with dates of coverage and contact with authors to identify additional sources), as well as the date the most recent search was executed.	6
Search	8	Present the full electronic search strategy for at least 1 database, including any limits used, such that it could be repeated.	54-56
Selection of sources of evidence†	9	State the process for selecting sources of evidence (i.e., screening and eligibility) included in the scoping review.	6-7
Data charting process‡	10	Describe the methods of charting data from the included sources of evidence (e.g., calibrated forms or forms that have been tested by the team before their use, and whether data charting was done independently or in duplicate) and any processes for obtaining and confirming data from investigators.	7
Data items	11	List and define all variables for which data were sought and any assumptions and simplifications made.	7
Critical appraisal of individual sources of evidence§	12	If done, provide a rationale for conducting a critical appraisal of included sources of evidence; describe the methods used and how this information was used in any data synthesis (if appropriate).	7-8

medRxiv preprint doi: https://doi.org/10.1101/2024.08.21.24312335; this version posted August 21, 2024. The copyright holder for this preprint (which was not certified by peer review) is the author/funder, who has granted medRxiv a license to display the preprint in perpetuity.
It is made available under a CC-BY 4.0 International license .

SECTION	ITEM	PRISMA-ScR CHECKLIST ITEM	REPORTED ON PAGE #
Synthesis of results	13	Describe the methods of handling and summarizing the data that were charted.	7
**RESULTS**			
Selection of sources of evidence	14	Give numbers of sources of evidence screened, assessed for eligibility, and included in the review, with reasons for exclusions at each stage, ideally using a flow diagram.	8, 57
Characteristics of sources of evidence	15	For each source of evidence, present characteristics for which data were charted and provide the citations.	8
Critical appraisal within sources of evidence	16	If done, present data on critical appraisal of included sources of evidence (see item 12).	31
Results of individual sources of evidence	17	For each included source of evidence, present the relevant data that were charted that relate to the review questions and objectives.	9, 13-15, 22-24
Synthesis of results	18	Summarize and/or present the charting results as they relate to the review questions and objectives.	10-12, 16-21, 25-30
**DISCUSSION**			
Summary of evidence	19	Summarize the main results (including an overview of concepts, themes, and types of evidence available), link to the review questions and objectives, and consider the relevance to key groups.	32-35
Limitations	20	Discuss the limitations of the scoping review process.	35-36
Conclusions	21	Provide a general interpretation of the results with respect to the review questions and objectives, as well as potential implications and/or next steps.	36
**FUNDING**			
Funding	22	Describe sources of funding for the included sources of evidence, as well as sources of funding for the scoping review. Describe the role of the funders of the scoping review.	3

From: Tricco AC, Lillie E, Zarin W, O'Brien KK, Colquhoun H, Levac D, et al. PRISMA Extension for Scoping Reviews (PRISMAScR): Checklist and Explanation. Ann Intern Med. 2018;169:467–473. doi: 10.7326/M18-0850.
